# Rifabutin-Based Rescue Therapy for *Helicobacter pylori* Eradication: A Long-Term Prospective Study in a Large Cohort of Difficult-to-Treat Patients

**DOI:** 10.3390/jcm8020199

**Published:** 2019-02-06

**Authors:** Davide Giuseppe Ribaldone, Sharmila Fagoonee, Marco Astegiano, Marilena Durazzo, Anna Morgando, Tatiana Sprujevnik, Chiara Giordanino, Monica Baronio, Claudio De Angelis, Giorgio Maria Saracco, Rinaldo Pellicano

**Affiliations:** 1 Department of Surgical Sciences, University of Turin, 10123 Turin, Italy; davrib_1998@yahoo.com; 2 Institute of Biostructure and Bioimaging (CNR) c/o Molecular Biotechnology Center, 10126 Turin, Italy; 3 Unit of Gastroenterology, Molinette Hospital, 10123 Turin, Italy; marcoastegiano58@gmail.com (M.A.); amorgando@cittadellasalute.to.it (A.M.); sprujevnik@yahoo.it (T.S.); chiaragiordanino@libero.it (C.G.); eusdeang@hotmail.com (C.D.A.); giorgiomaria.saracco@unito.it (G.M.S.); 4 Department of Medical Sciences, University of Turin, 10123 Turin, Italy; marilena.durazzo@unito.it; 5 Unit of Gastroenterology, Maria Vittoria Hospital, 10100 Turin, Italy; monica.baronio@aslto2.piemonte.it

**Keywords:** rifabutin, *Helicobacter pylori*, gastritis, peptic ulcer, dyspepsia, gastric cancer

## Abstract

The most commonly used regimens fail to eradicate *Helicobacter pylori* (*H. pylori*) infection in 5–10% of patients. Those not cured with treatments based on amoxicillin, clarithromycin, nitroimidazoles, fluoroquinolones, bismuth or tetracycline have no other conventional options thereafter. In this prospective long-term monocentric study, patients who failed to eradicate *H. pylori* following treatment with all conventional antibiotics were included. All subjects were treated with rifabutin 150 mg, amoxicillin 1 g and a standard dose of proton pump inhibitor, twice daily for 14 days. A negative ^13^C-urea breath test was used four weeks after treatment completion as an index of *H. pylori* eradication. Three hundred and two patients were included. Fifty-four percent (164/302) had peptic ulcer disease while 45.7% (138/302) had gastritis or functional dyspepsia. Per-protocol eradication and intention-to-treat eradication were achieved in 72.7% and 71.5%, respectively. A univariate analysis showed that gender, ethnic background, smoking habits and familial history of gastric diseases were not predictive factors of response, while with multiple logistic regression analysis, the ethnic background (Italian) predicted a poor response in the second period of the study (2010–2017). In conclusion, this study on a large cohort of very difficult-to-treat patients showed that rifabutin-based rescue therapy is an acceptable and safe strategy after multiple eradication failures with conventional antibiotics.

## 1. Introduction

The bacterium *Helicobacter pylori* (*H. pylori*) is recognized as the most important cause of gastritis and peptic ulcer (PU) and a risk factor for gastric cancer (GC). Presently, only 10% of subjects with a history of PU have relapses after *H. pylori* eradication [1]. Moreover, in recent decades, several studies have reported on the potential link between chronic *H. pylori* infection and a variety of extra-gastroduodenal manifestations [2].

Despite efforts aimed at optimizing *H. pylori* treatment, the ideal modality to eradicate this infection with a simple regimen is still lacking. This is mainly due to increasing antibiotic resistance, especially to clarithromycin, as reported by a multicenter European study [3] and other data worldwide [4]. The Maastricht V/Florence Consensus Report of the European Helicobacter and Microbiota Study Group has advised to use a clarithromycin-based triple therapy as the first choice in treating *H. pylori* infection after the clarithromycin resistance rate of each region has been considered [5]. Alternative schedules and approaches are available, such as testing for clarithromycin resistance [6], prescribing proton pump inhibitors (PPIs), amoxicillin, clarithromycin and metronidazole concomitantly or using the bismuth-based quadruple therapy with its “three in one” formulation [7]. Fluoroquinolones are the most employed drugs for rescue therapy after failure of previous treatments [1,5]. However, even with the most commonly used treatment regimens, approximately 5–10% of patients fail to eradicate *H. pylori* infection [1]. Patients not cured with treatments including clarithromycin, nitroimidazoles, fluoroquinolones, bismuth and tetracycline and, recently, the “three in one” pills, had no other logical empirical treatment thereafter.

Rifabutin-based rescue therapy has become a promising alternative after several *H. pylori* eradication failures [8]. This bacterium was found to be highly susceptible in vitro to rifabutin, an anti-mycobacterial agent and a spiropiperidyl derivative of rifamycin S [9]. Furthermore, the number of *H. pylori* strains that remained resistant to rifabutin was low when tested under experimental conditions. In a clinical setting, until now, 0–46.1% of rifabutin-resistant strains have been isolated from patients who were either treated or not for *H. pylori* infection [10]. 

In the present 17-year prospective study, we aimed to evaluate the efficacy and tolerability of a rifabutin-based regimen in a cohort of patients with four prior consecutive *H. pylori* eradication failures.

## 2. Materials and Methods

The study was conducted at the Outpatient Clinic, Unit of Gastroenterology, Molinette and San Giovanni Antica Sede Hospitals, Turin, Italy, from June 2000 to June 2017.

We prospectively enrolled all consecutive patients who failed to eradicate *H. pylori* infection after treatment with four regimens, including clarithromycin, amoxicillin, bismuth, tetracycline, metronidazole and levofloxacin. Since our outpatient clinic is a reference center for all hospitals and outpatient clinics of Piedmont Region, Northwest of Italy [11], the patients included in this study were brought to our attention by generalists as well as several Gastroenterology units.

Exclusion criteria included any known allergy to rifabutin or rifamycin, previous gastric surgery, presence of associated conditions (pregnancy or feeding) or comorbidities (hepatic, cardiorespiratory or renal diseases, malignancies or coagulopathy) which limited rifabutin administration. 

The investigation was carried out following the rules of the Declaration of Helsinki of 1975, revised in 2013. Since the prescription of rifabutin in this context is considered "off-label" in Italy, the study was approved by the Molinette Hospital Pharmacy and Therapeutics Committee (Project identification code 29.01.2009). Written informed consent was obtained from all patients prior to treatment.

*H. pylori* infection was assessed by histology or by the ^13^C-urea breath test (^13^C-UBT), performed according to the supplier’s instructions (Expirobacter®, Sofar, Trezzano Rosa, Italy). Samples were analyzed for ^13^C/^12^C ratio with a mass spectrometer (BreathMAT plus, Finnigan, Bremen, Germany). Results were expressed as excess ð^13^CO_2_ excretion per milliliter: A value >4 delta per mil was considered positive. No patient received PPIs or antibiotics in the preceding 30 days. 

All patients were treated with rifabutin 150 mg, amoxicillin 1 g, and a PPI (omeprazole 20 mg, esomeprazole 40 mg, pantoprazole 40 mg, rabeprazole 40 mg, or lansoprazole 30 mg), twice daily for 14 days. Adherence to rifabutin intake was assessed via patient self-report. Each patient was visited in the week after treatment to have their general health status evaluated. Self-reported adverse events were also recorded during this visit. 

In all patients, post-therapy *H. pylori* status was assessed and the bacterium was considered eradicated upon a negative ^13^C-UBT taken 4 weeks after the treatment was completed.

### Statistical Analysis

For quantitative variables, mean and standard deviation (SD) were calculated. For qualitative variables, percentage and 95% confidence interval (CI) were provided. The Chi-squared test was used to analyze the categorical variables. Analysis of *H. pylori* eradication efficacy was performed on an ‘intention-to-treat’ (ITT) basis (including all eligible patients enrolled in the study regardless of compliance with the protocol) and on a ‘per-protocol’ (PP) basis (excluding patients who had consumed less than 90% of the medication provided and those with unevaluable data after therapy). 

Thereafter, a multiple logistic regression analysis was carried out to calculate adjusted odds ratios (ORs) for age, gender, ethnic background, *H. pylori* associated disease, smoking habits, and familial history of GC. First, each variable was entered individually, then all the variables were analyzed together (full model). Hosmer-Lemeshow statistics were used to assess the model fit. For each covariate, the ORs and 95% CI were reported. The statistical analysis was performed with MedCalc Statistical Software version 14.8.1 (MedCalc Software bvba, Ostend, Belgium; http://www.medcalc.org; 2014).

## 3. Results

Three hundred and two patients fulfilled the above reported criteria and were included in the study. Demographic and clinical details are listed in Table 1. Regarding the ethnic background, 56 patients were non-Italian. These included 19 Romanians, 15 Peruvians, 11 Moroccans, 3 Egyptians, 2 Moldavians, 2 Ukrainians, 1 Pole, 1 Filipino, 1 Mongolian, and 1 Iranian. 

All but five patients were compliant with the medication period (i.e., completed the entire period of planned treatment). The five patients were non-compliant because of adverse events in four subjects and the fifth patient reconsidered after signing the consent.

Per-protocol eradication was achieved in 216/297 (72.7%, 95%CI: 67.3%–77.7%) patients. Intention-to-treat eradication was achieved in 216/302 (71.5%, 95%CI: 66.1%–76.5%) patients.

To search for potential prognostic factors of poor response, we conducted a univariate analysis (Supplementary Table S1). When considering gender, ITT eradication was achieved in 132/183 (72.1%, 95%CI: 65%–78.5%) females, and in 84/119 (70.6%, 95%CI: 61.5%–78.6%) males (*p* = 0.87). When classified according to ethnic background, ITT eradication was achieved in 175/246 (71.1%, 95%CI: 65%–76.7%) Italians, and in 41/56 (73.2%, 95%CI: 59.7%–84.2%) foreigners (*p* = 0.88). When smoking habits were considered, ITT eradication was achieved in 75/102 (73.5%, 95%CI: 63.9%–81.8%) active smokers, 42/63 (66.7%, 95%CI: 53.7%–78%) ex-smokers and in 99/137 (72.3%, 95%CI: 64%–79,6%) never-smokers (*p* > 0.44). Among patients with known familial anamnesis, ITT eradication was achieved in 67/93 (72%, 95%CI: 61.8%–80.9%) patients with a family history of gastritis, PU or GC, and in 135/191 (70.7%, 95%CI: 63.7%–77%) of subjects without a family history of gastritis, PU or GC (*p* = 0.92).

Since the location of our Outpatient Clinic changed in June 2010, in a sub analysis, we divided two group of patients: those treated in the period from June 2000 to June 2010, and those treated in the period from July 2010 to June 2017. In the first period, PP eradication was obtained in 137/173 (79.2%, 95%CI: 72.4%–85%) patients while ITT eradication was achieved in 137/176 (77.8%, 95%CI: 71%–83.7%) patients. In the second period, PP eradication was obtained in 79/124 (63.7%, 95%CI: 54.6%–72.2%) patients while ITT eradication was achieved in 79/126 (62.7%, 95%CI: 53.6%–71.1%) patients. The ITT eradication rate in the group of patients treated in the last seven years decreased significantly by 15.1% (95%CI: 4.3%–26%; *p* = 0.006).

To further determine whether the reduction in PP and ITT eradications in the second period could be caused by a change in population features, age, gender, ethnic background, *H. pylori* associated disease, smoking habits, and familial history of GC were considered. Importantly, Italian patients decreased from 85.8% (151/176) in the first period to 75.4% (95/126) in the second period (*p* = 0.03), and the reduction in eradication rate occurred more prominently among Italians (from 117/151, 77.5% to 58/95, 61.1%, reduction of 16.4%, *p* = 0.009) than among foreigners (from 20/25, 80% to 21/31, 67.7%, reduction of 12.3%, *p* = 0.46). The percentage of active smokers, ex-smokers, and never-smokers did not change between the first period (57/176, 32.4%, 44/176, 25%, 75/176, 42.6%) and the second period (45/126, 35.7%, 19/126, 15.1%, 62/126, 49.2%) (*p* > 0.31). The proportion of males also remained unaltered between the first period (70/176, 39.8%) and the second period (49/126, 38.9%) (*p* = 0.97). Among patients with a known familial anamnesis, the proportion of those with a family history of gastritis, PU or GC significantly increased between the first (44/161, 27.3%) and the second (49/123, 39.8%) period (*p* = 0.036). In the first period, ITT eradication was achieved in 34/44 (77.2%) patients with a family history of gastritis, PU or GC, and in 92/117 (78.6%) patients without a family history of gastritis, PU or GC *(p=* 0.90). In the second period, ITT eradication was achieved in 33/49 (67.3%) patients with a family history of gastritis, PU or GC, and in 43/74 (58.1%) patients without a family history of gastritis, PU or GC (*p* = 0.4) (Supplementary Table S2). 

The multiple logistic regression analysis for *H. pylori* eradication rate, controlled for the single variable or all variables, did not reveal any significant association (Supplementary Table S3). However, when verified for change in population features, the decrease in Italian ethnic background was statistically associated with a decrease in efficacy of rifabutin treatment; when controlling for all variables, the ORs for the decrease in Italian ethnic background resulted in 0.799 (*p* = 0.02) (Supplementary Table S4).

Adverse effects were reported in 22/302 (7.3%) cases, and included abdominal/epigastric pain (*n* = 9), nausea/vomiting (*n* = 7), diarrhea (*n* = 2), fatigue (*n* = 1), headache (*n* = 1) and oral candidiasis (*n* = 1). Furthermore, allergy occurred in one case. Since one patient with abdominal pain reported fever, blood cell count was performed to exclude myelotoxicity. The results were similar to those obtained eight months earlier before the beginning of the rifabutin treatment. Four patients opted out of treatment due to adverse effects: in two cases for severe epigastric pain that disappeared seven days after rifabutin suspension without biochemical or abdominal ultrasonographic alterations, in one case for allergy, and in another case for severe fatigue without weight loss.

It is interesting to highlight that one patient, treated in 2017, suffered from acquired immune deficiency syndrome (AIDS), stage 2, under treatment with ritonavir and dolutegravir. Therapy with rifabutin was undertaken after authorization from infectious disease specialists. The patient regularly ended the rifabutin-based treatment and eradicated *H. pylori* without alteration in the usual blood cell count.

## 4. Discussion

The utility of *H. pylori* culture, with antibiotic susceptibility testing, remains of high importance to define a tailored therapy [5]. However, culture is not always available on a routine basis [12]. On the other hand, it is not helpful to repeat any of the unsuccessful antibiotic treatment schemes already used by the patients [13]. Thus, when a different antibiotic against *H. pylori*-resistant strains becomes available, in a context in which antibiotic susceptibility testing is not accessible, the former may be prescribed without performing bacterial culture; in particular in cases where regimens based on clarithromycin, amoxicillin, bismuth, tetracycline, metronidazole and levofloxacin have failed [9]. In this context, rifabutin-based rescue therapies have become an encouraging strategy for circumventing eradication failures, although an increasing *H. pylori* resistance rate has been reported [10]. In the present study, the ITT eradication rate of around 70% achieved with the combination of rifabutin-amoxicillin-PPI for 14 days is encouraging, considering the history of several eradication failures and the lack of alternatives in this cohort. Our data also raised some concern about the decreased eradication rate in the two analyzed periods. Among several variables analyzed, such as smoking habits, family history of gastric diseases and gender, none were associated with this change. Of note, the decrease of the proportion of Italian patients treated between the first and the second period was statistically significant (*p* = 0.03), and the reduction of the eradication rate was more prominent among Italians than foreigners.

Several authors have reported encouraging results with rifabutin in the treatment of patients failing to eradicate *H. pylori* infection after several attempts [14,15,16]. Importantly, in a systematic review with meta-analysis, Gisbert and Calvet reported data from May 2011, on the eradication rate using rifabutin-based regimens, in several studies including 1008 patients with multiresistant *H. pylori*. Overall, the mean *H. pylori* eradication rate (ITT analysis) with rifabutin-containing regimens was 73% (95%CI: 67%–79%). Cure rates for second-line, third-line and fourth/fifth-line rifabutin therapies were 79% (67%–92%), 66% (55%–77%) and 70% (60%–79%), respectively [9]. Our results, obtained from a large cohort of patients at the fifth-line regimen, are in agreement with these data. Recently, new rifabutin-based regimens have been proposed. Ciccaglione et al. reported on a small sample size population that the addition of bismuth subcitrate to a triple therapy that included PPIs, amoxicillin, and rifabutin in patients treated for the third time for *H. pylori* infection, resulted in 30% therapeutic gain compared to rifabutin-based triple therapy alone [17].

Several concerns still remain regarding rifabutin treatment. This drug is expensive and sometimes, severe leukopenia and thrombocytopenia have been reported, with reversible myelotoxicity demonstrated by bone marrow aspirate [15]. In our study, no patient, including the one affected by AIDS, developed myelotoxicity. Another concern about the widespread use of rifabutin for *H. pylori* eradication regards the potential increase in rate of resistance to multiple strains of *Mycobacterium tuberculosis*. Hence, rifabutin should be restricted to patients who have experienced multiple failures of *H. pylori* therapies. Some authors have reported, with multistep strategies, a final eradication rate of almost 100% [18,19]. This emphasizes the recommendation that local antibiotic resistance or local clinical results should be considered when designing a treatment strategy against *H. pylori* infection. In this respect, a wider perspective of the benefits of treating again for *H. pylori* infection can be obtained if cumulative eradication rates, not only absolute figures, with successive re-treatments are taken into account. 

Resistance to rifabutin exists [20]. Rifamycin targets the DNA-directed RNA polymerase and primarily the ß-subunit encoded by the *rpoB* gene. Recently, Hays et al. reported the molecular characterization of *H. pylori* resistance to rifabutin. By using rifampicin to screen for rifabutin resistance in vitro, the authors sequenced the *rpoB* gene. All 54 resistant strains carried at least one mutation in the *rpoB* gene at codons 525, 530, 538, 540 in the rifampicin resistance determining region. A new mutation, L547, responsible for resistance to rifamycins, was also reported [21].

The strengths of our study include the large sample size (to our knowledge, the largest among those published hitherto) and the long-term study period. The fact that all patients were at the same step of treatment permits a conclusion on a very difficult-to-treat population. In fact, while most studies have used rifabutin as a second or a third option, we have used this drug after four prior treatments. All patients in this study failed to eradicate *H. pylori* with a clarithromycin-based treatment, with a metronidazole non-bismuth treatment, with a quadruple bismuth-treatment (with metronidazole and tetracycline) and with a levofloxacin-based treatment. However, we could not perform antimicrobial susceptibility testing prior to administration of these study drugs due to the fact that this test was not available on a routine basis in the study location. On the other hand, as far as *H. pylori*-rifabutin resistance is concerned, the role of the culture-guided treatment remains unclear [22]. 

Several limitations of this study should be highlighted. First, the self-report strategy used to assess adherence to therapy. This approach could be limited by poor accuracy of assessment and debatable precision. Second, due to an exclusive clinical strategy, not supported by susceptibility testing, there is a lack of resistance data. Thus, the causes (lack of adherence to therapy or antibiotic resistance surge) of preceding treatment failures in these patients are not available. Third, due to some features of our study design, there was no real homogeneity in the study population. In particular, patients treated with different PPIs over time, a suboptimal diagnostic method used in some cases and the long study duration could have led to an uneven distribution of ethnic background, resulting in an uneven distribution of drug resistance pattern. New strategies to cure *H. pylori* infection have most recently become available. Considering the current revitalization of bismuth [23], the results of the new drug formulation with bismuth subcitrate potassium, metronidazole, and tetracycline contained in a single capsule (three-in-one) are indeed promising. Previous studies have shown that this formulation (three tablets taken four times a day and a PPI taken separately twice a day) for 10 days is a suitable salvage therapy for patients who failed clarithromycin-based triple therapy [7]. Whether this approach would reduce the need for treatment with rifabutin remains to be explored. Similarly, it will be interesting evaluate in randomized trials the efficacy of the three-in-one regimen in comparison with the rifabutin-based therapy in difficult-to-treat patients. Nevertheless, it could be hypothesized that the actual diffuse use as first-line treatment of the former will favorably change the failure rates, thus reducing the number of patients requiring rifabutin.

## 5. Conclusions

In conclusion, rifabutin triple therapy is an acceptable, alternative empiric treatment for patients with multiple treatment failures when the antimicrobial susceptibility testing is not available. In the future it will be important to evaluate whether the efficacy of this regimen might decrease on a yearly basis. 

## Figures and Tables

**Table 1:** Characteristics of the population.

Clinical Characteristics	Distribution
**Whole population, *n***	302
**Mean age ± SD**	57 ± 13
**Gender**	
Male, *n* (%)	135 (44.7%)
Female, *n* (%)	167 (55.3%)
**Ethnic background**	
Italian, *n* (%)	246 (81.5%)
Non-Italian, *n* (%)	56 (18.5%)
***H. pylori* associated disease**	
PU disease, *n* (%)	164 (54.3%)
Bleeding, *n* (%)	28 (9.3%)
Perforation, *n* (%)	5 (1.7%)
**Gastritis or functional dyspepsia**	138 (45.7%)
**Familial history of GC**	56 (18.5%)

Data are mean ± SD. SD, standard deviation; *H. pylori, Helicobacter pylori;* PU, peptic ulcer; GC, gastric cancer.

## Supplementary Materials

The supplementary materials are available online at http://www.mdpi.com/2077-0383/8/2/199/s1.

## References

[1] Pellicano R., Ribaldone D.G., Fagoonee S., Astegiano M., Saracco G.M., Mégraud F. (2016). A 2016 panorama of *Helicobacter pylori* infection: Key messages for clinicians. Panminerva. Med..

[2] De Korwin J.-D., Ianiro G., Gibiino G., Gasbarrini A. (2017). *Helicobacter pylori* infection and extragastric diseases in 2017. Helicobacter.

[3] Mégraud F., Coenen S., Versporten A., Kist M., Lopez-Brea M., Hirschl A.M., Andersen L.P., Goossens H., Glupczynski Y., on behalf of the Study Group participants (2013). *Helicobacter pylori* resistance to antibiotics in Europe and its relationship to antibiotic consumption. Gut.

[4] Buzàs G.M. (2018). Benign and malignant gastroduodenal diseases associated with *Helicobacter pylori*: A narrative review and personal remarks in 2018. Minerva. Gastroenterol. Dietol..

[5] Malfertheiner P., Mégraud F., O’Morain C.A., Gisbert J.P., Kuipers E.J., Axon A.T., Bazzoli F., Gasbarrini A., Atherton J., Graham D.Y. (2017). Management of *Helicobacter pylori* infection-the Maastricht V/Florence Consensus Report. Gut.

[6] Cosme A., Montes M., Ibarra B., Tamayo E., Alonso H., Mendarte U., Lizasoan J., Herreros-Villanueva M., Bujanda L. (2017). Antimicrobial susceptibility testing before first-line treatment for *Helicobacter pylori* infection in patients with dual or triple antibiotic resistance. World J. Gastroenterol..

[7] Malfertheiner P., Bazzoli F., Delchier J.C., Celiñski K., Giguère M., Rivière M., Mégraud F., Pylera Study Group (2011). *Helicobacter pylori* eradication with a capsule containing bismuth subcitrate potassium, metronidazole, and tetracycline given with omeprazole versus clarithromycin-based triple therapy: A randomised, open-label, non-inferiority, phase 3 trial. Lancet.

[8] Nishizawa T., Suzuki H., Matsuzaki J., Muraoka H., Tsugawa H., Hirata K., Hibi T. (2011). *Helicobacter pylori* resistance to rifabutin in the last 7 years. Antimcrob. Agents Chemother..

[9] Gisbert J.P., Calvet X. (2012). Review article: Rifabutin in the treatment of refractory *Helicobacter pylori* infection. Aliment. Pharmacol. Ther..

[10] O’Connor A., Lamarque D., Gisbert J., O’Morain C. (2017). Treatment of *Helicobacter pylori* infection 2017. Helicobacter.

[11] Ribaldone D.G., Mazzucco D., Fagoonee S., Crocellà L., Lavagna A., Fracchia M., Caviglia G.P., Simondi D., Rocca R., Astegiano M. (2018). Management of *Helicobacter pylori* in Piedmont, Italy. Minerva. Gastroenterol. Dietol..

[12] Costa S., Soares J.B., Gonçalves R. (2017). Efficacy and tolerability of culture-guided treatment for *Helicobacter pylori* infection. Eur. J. Gastroenterol. Hepatol..

[13] Huang J.Q., Hunt R.H. (1999). Treatment after failure: The problem of non-responders. Gut.

[14] Perri F., Festa V., Clemente R., Quitadamo M., Andriulli A. (2000). Rifabutin-based “rescue therapy” for *Helicobacter pylori* infected patients after failure of standard regimens. Aliment. Pharmacol. Ther..

[15] Canducci F., Ojetti V., Pola P., Gasbarrini G., Gasbarrini A. (2001). Rifabutin-based *Helicobacter pylori* eradication “rescue therapy”. Aliment. Pharmacol. Ther..

[16] Gisbert J.P., Castro-Fernandez M., Perez-Aisa A., Cosme A., Molina-Infante J., Rodrigo L., Modolell I., Cabriada J.L., Gisbert J.L., Lamas E. (2012). Fourth-line rescue therapy with rifabutin in patients with three *Helicobacter pylori* eradication failures. Aliment. Pharmacol. Ther..

[17] Ciccaglione A.F., Tavani R., Grossi L., Cellini L., Manzoli L., Marzio L. (2016). Rifabutin containing triple therapy and rifabutin with bismuth containing quadruple therapy for third-line treatment of *Helicobacter pylori* infection. Two pilot studies. Helicobacter.

[18] Gasbarrini A., Ojetti V., Armuzzi A., Branca G., Canducci F., Torre E.S., Candelli M., Pastorelli A., Anti M., Fedeli G., Fadda G., Pola P., Gasbarrini G. (2000). Efficacy of a multi step strategy for *Helicobacter pylori* eradication. Aliment. Pharmacol. Ther..

[19] Gisbert J.P., Gisbert J.L., Marcos S., Jimenez-Alonso I., Moreno-Otero R., Pajares J.M. (2008). Empirical rescue therapy after *Helicobacter pylori* treatment failure: A 10-year single-centre study of 500 patients. Aliment. Pharmacol. Ther..

[20] Heep M., Odenbreit S., Beck D., Decker J., Prohaska E., Rieger U., Lehn N. (2000). Mutations of four distinct regions of the rpoB gene can reduce the susceptibility of *Helicobacter pylori* to rifamycins. Antimicrob. Agents Chemother..

[21] Hays C., Burucoa C., Lehours P., Tri Tran C., Leleu A., Raymond J. (2017). Molecular characterization of *Helicobacter pylori* resistance to rifamycins. Helicobacter.

[22] Gisbert J.P., Pajares J.M. (2005). *Helicobacter pylori* rescue therapy after failure of two eradication treatments. Helicobacter.

[23] Actis G.C. (2017). *Helicobacter pylori* 2017: Revitalized therapies for an ever-challenging bug. Panminerva. Med..

