# Supplementary material for: Rifabutin-Based Rescue Therapy for Helicobacter pylori Eradication: A Long-Term Prospective Study in a Large Cohort of Difficult-to-Treat Patients
Source: J Clin Med. 2019 Feb 6;8(2):199. doi: 10.3390/jcm8020199 (PMC6406425; doi:10.3390/jcm8020199)
Supplement: Supplementary file 1 [file jcm-08-00199-s001.pdf]

Journal of
Clinical Medicine

MDPI

Supplementary Table S1. Eradication rate in the whole period.

	Eradication Rate	*p*-value
**Whole population**	PP = 72.7% (216/297)<br>ITT = 71.5% (216/302)	
**Gender**	Males = 70.6% (84/119)<br>Females = 72.1% (132/183)	0.87
**Ethnic background**	Italians = 71.1% (175/246)<br>Foreigners = 73.2% (41/56)	0.88
**Smoking habits**	Active smokers = 73.5% (75/102)<br>Ex-smokers = 66.7% (42/63)<br>Never-smokers = 72.3% (99/137)	> 0.44
**Family history (if known) GC**	Positive = 72% (67/93)<br>Negative = 70.7% (135/191)	0.92

PP = Per-protocol; ITT = Intention-to-treat eradication; GC = gastric cancer

Supplementary Table S2. Eradication rate according to decade.

**Treatment period**	**June 2000-June 2010**	**July 2010-June 2017**	***p*-value**
**Whole population**	ER = 77.8% (137/176)	ER = 62.7% (79/126)	0.006
**Ethnic background**			
• Italians	ER = 77.5% (117/151)	ER = 61.1% (58/95)	0.009
• Foreigners	ER = 80% (20/25)	ER = 67.7% (21/31)	0.46
**Smoking habits**			
• active smokers	NC = 32.4% (57/176)	NC = 35.7% (45/126)	>0.31
• ex-smokers	NC = 25% (44/176)	NC = 15.1% (19/126)	
• never-smokers	NC = 42.6% (75/176)	NC = 49.2% (62/126)	
**Sex**			
• male	NC = 39.8 (70/176)	NC = 38.9% (49/126)	0.97
• female	NC = 60.2 (106/176)	NC = 61.1% (77/126)	
**Family history (if known) of GC**			
• Positive	ER = 77.2% (34/44)	ER = 67.3% (33/49)	0.9
• Negative	ER = 78.6% (92/117)	ER = 58.1 % (43/74)	0.4

ER = eradication rate; NC = number of cases; GC = gastric cancer

Supplementary Table S3. Multiple logistic regression analysis for eradication rate controlling for several potential confounders.

	Single variables			All variables		
**Covariates**	**OR**	**95%CI of OR**	***p*- value**	**ORs**	**95%CI of OR**	***p*- value**
**Age**	1.001	0.997- 1.005	0.608	0.989	0.971- 1.009	0.283
**Gender**						
Men	1.000	-	-	1.000	-	-
Women	1.015	0.877- 1.279	0.870	1.097	0.805- 1.501	0.930
**Ethnic background**						
Italians	1.000	-	-	1.000	-	-
Foreigners	1.019	0.855- 1.302	0.880	1.125	0.798- 1.683	0.940
***H. pylori* associated disease**						
PU disease	1.000	-	-	1.000	-	-
Bleeding	0.902	0.932- 1.299	0.810	0.854	0.786- 1.451	0.890
Perforation	N/A			N/A		
Gastritis or functional dyspepsia	1.103	0.956- 1.199	0.820	1.090	0.905- 1.297	0.880
**Smoking history**						
Active smokers	1.000	-	-	1.000	-	-
Ex-smokers	0.846	0.760- 1.307	0.640	0.903	0.811- 1.436	0.760
Never-smokers	1.286	0.860- 1.407	0.440	1.120	0.711- 1.436	0.560
**Family history (if known) GC**						
No	1.000	-	-	1.000	-	-
Yes	0.933	0.903- 1.122	0.920	0.956	0.893- 1.368	0.940

OR = odds ratio; CI = confidence interval; *H. pylori* = *Helicobacter* pylori; PU = peptic ulcer;
N/A = not applicable due to too low sample size; GC = gastric cancer

Supplementary Table S4. Multiple logistic regression analysis for eradication rate controlling for change in population features.

	Single variables			All variables		
**Covariates**	**OR**	**95%CI of OR**	***p*- value**	**ORs**	**95%CI of OR**	***p*- value**
**Age variation** +2 ± 4 years	0.967	0.937- 1.105	0.518	0.889	0.851- 1.209	0.728
**Gender variation**						
Men + 3 ± 2%	0.983	0.954- 1.041	0.999	0.954	0.909- 1.095	0.990
**Ethnic background variation**						
Italians -10.4 ± 4.3%	0.780	0.569- 0.899	0.005	0.799	0.502- 0.901	0.020
***H. pylori* associated disease variation**						
PU disease -2.3 ± 0.9%	1.000	-	-	1.000	-	-
Bleeding -1.9 ± 0.8%	0.932	0.892- 1.239	0.840	0.894	0.826- 1.351	0.780
Perforation	N/A			N/A		
Gastritis or functional dyspepsia +4.2 ± 1.9%	1.145	0.905- 1.259	0.891	1.097	0.805- 1.397	0.910
**Smoking history variation**						
Active smokers -2.0 ± 0.9%	1.000	-	-	1.000	-	-
Ex-smokers +1.2 ± 0.8%	0.946	0.870- 1.237	0.656	0.803	0.699- 1.566	0.870
Never-smokers +0.8 ± 0.7%	1.268	0.956- 1.079	0.440	1.102	0.911- 1.136	0.470
**Family history (if known) GC variation**						
Yes	0.943	0.907- 1.134	0.912	0.976	0.978- 1.168	0.950

OR = odds ratio; CI = confidence interval; *H. pylori* = *Helicobacter* pylori; PU = peptic ulcer; N/A = not applicable due to too low sample size; GC = gastric cancer
